# Relevance of improved reporting for non-pharmacological interventions in clinical trials: Bridging research to clinical practice

**DOI:** 10.1016/j.bjpt.2026.101653

**Published:** 2026-09-12

**Authors:** Francisco Alburquerque-Sendín, Daiana Priscila Rodrigues-de-Souza

**Affiliations:** a Department of Nursing, Pharmacology and Physical Therapy, University of Cordoba, Cordoba, Spain; b Maimonides Institute for Biomedical Research of Cordoba (IMIBIC), Reina Sofia University Hospital, University of Cordoba, Cordoba, Spain

**Keywords:** Intervention description, Intervention fidelity, Reproducibility, Evidence-based practice

## Abstract

**What is known:**

Accurate description and fidelity of non-pharmacological interventions are critical to translating research findings into clinical practice. The lack of such detailed reporting undermines the essential and stable connection between research and clinical practice.

**What should be known:**

The aim of the current masterclass is to discuss how to improve the quality of reporting and reproducibility in non-pharmacological interventions.

**What should change:**

Enhance the quality requirements for describing non-pharmacological interventions in the prospective registration of clinical trials. Mandate the use of the Template for Intervention Description and Replication (TIDieR) and Consensus on Exercise Reporting Template (CERT) checklists, and of fidelity evaluation for all clinical trials involving non-pharmacological interventions. These requirements should extend from clinical trial registration through subsequent publications, including grant applications, protocols, abstracts, and preprints. Ensure that systematic reviews and clinical practice guidelines apply not only general methodological criteria but also intervention-specific criteria and context for non-pharmacological interventions, respectively, to guarantee reproducibility and achieve expected outcomes.

**What to expect:**

In the short term, improving the description and traceability of non-pharmacological interventions may encounter resistance to change. However, greater rigor should not be avoided, even if it results in a temporary reduction in the volume of available scientific evidence. Ultimately, prioritizing quality over quantity in healthcare research may lead to more meaningful and impactful advancements in clinical practice.

## Highlights

• Incomplete intervention reporting makes effective treatments hard to reproduce in clinical practice.
• Registries, journals, and reviews should require the application of intervention description and fidelity‑assessment tools.
• Systematic reviews and guidelines must appraise intervention reporting and contextual feasibility, not only risk of bias.
• Improving the traceability and fidelity of interventions may temporarily reduce publication volume but ultimately strengthen clinical applicability.

## Clinicians cannot implement what trials do not describe

**Fig. 1:**
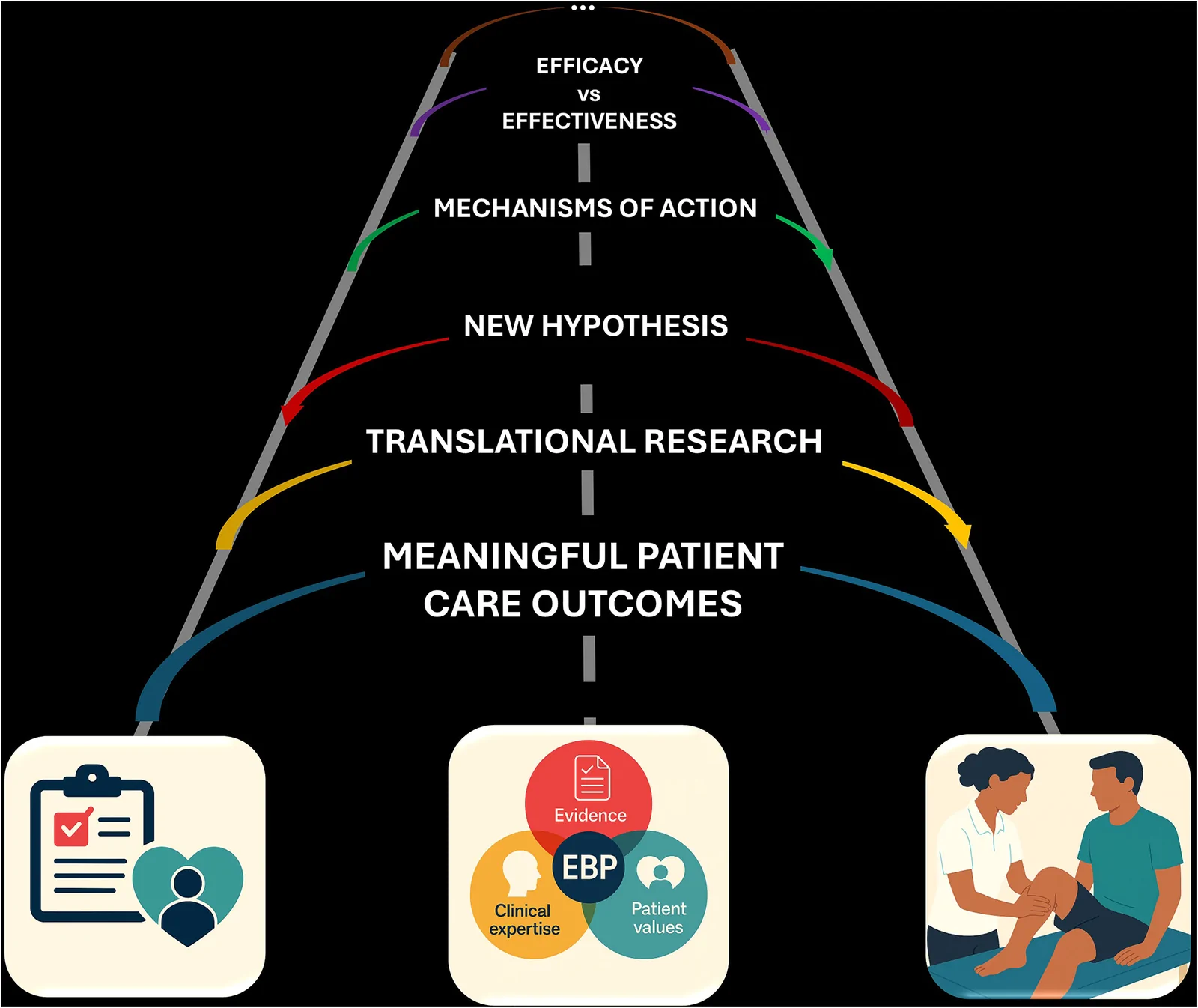
Bridging Research domain and Clinical Practice. The left line represents the Research domain; the dashed line represents Evidence-based practice (EBP); the right line represents Clinical practice. Only a limited subset of bridges is illustrated.

The model of evidence‑based practice, formulated over three decades ago,^1^ highlights the need to integrate high‑quality research, clinical expertise, and patient values in order to achieve meaningful patient care outcomes.^2^ Within this paradigm, research and clinical practice are expected to interact continuously: research generates knowledge intended to improve health outcomes, while clinicians apply that knowledge in individualized real‑world contexts.^2^ Although research and clinical practice share the common goal of improving patient health, research aims to establish internal validity and control sources of bias, whereas clinical practice must navigate variability in patient characteristics, practitioner expertise, available resources, and organizational demands. Consequently, the two domains progress in parallel rather than fully overlapping (Fig. 1), which does not imply disconnection.^3^^,^^4^

A central element that supports this coordination is methodological research rigor. When methodological issues arise, such as incomplete descriptions of non-pharmacological interventions, insufficient information about implementation processes, or a lack of clarity regarding fidelity, clinicians cannot reproduce the interventions in clinical practice.^5^ In the context of non-pharmacological interventions, which are often complex, individualized, and context dependent, insufficient detail about the *what, how, why*, and *who* of an intervention introduces avoidable barriers to implementation. Consequently, the relationship between research and practice depends not only on the strength of evidence but also on the traceability and clarity of the intervention itself. Therefore, the aim of the current masterclass is to discuss how to improve the quality of reporting and reproducibility in non-pharmacological interventions.

## Interventions are the core of clinical trials

More than 15 years ago, it was documented that complex interventions were often poorly described in clinical trials.^6^ More recently, a similar lack of detail has been observed in the reporting of exercise interventions.^7^ To address these issues, several initiatives have been proposed.

The first initiative concerned the integrity of the various components involved in the development and reporting of clinical trials, as outlined in the CONSORT statement. This statement, now over 30 years old, is a reporting guideline that specifies minimum items to include when reporting randomized trials.^8^ In 2017, CONSORT introduced a major adaptation for non-pharmacological interventions,^9^ mainly in the *Intervention* section, described as *Precise details of both the experimental treatment and comparator*, which included four additional topic items (5a-d). Other aspects of the CONSORT extension related to the description and fidelity of interventions span nearly all sections of clinical trial reports (items: 3a, 4a, 7a, 11a, 11b, 13new, 15, 20, 21), apart from the *Introduction* section.^9^ These items highlight the importance of intervention information in non-pharmacological trials. Nevertheless, this effort alone is not sufficient to ensure a thorough description or an accurate evaluation of the interventions.

The second initiative aimed to identify what would be done for each clinical trial before it began. Beyond the mandatory requirement for a priori approval by a research ethics committee, this process involves public disclosure of the trial’s topic, design, study population, and the interventions to be implemented, as well as any modifications to these elements throughout the trial’s execution. Accordingly, the prospective registration of clinical trials has become a standard prerequisite for publication in scientific journals. Since 2005, the International Committee of Medical Journal Editors (ICMJE) has mandated that clinical trials be registered in a publicly accessible registry at or before the enrollment of the first participant, as a condition for manuscript consideration.^10^ This policy has been reinforced by other global initiatives such as the WHO’s International Clinical Trials Registry Platform,^11^ that aggregate interventional and observational trial records and report counts by year, country and region.^12^ Despite the benefits of prospective trial registration, a careful and detailed evaluation is needed regarding the extent to which registration requirements are fulfilled.

The third initiative focused on enhancing the description of non-pharmacological interventions. To address this gap, the Template for Intervention Description and Replication (TIDieR)^13^ and the Consensus on Exercise Reporting Template (CERT)^14^ guidelines, both in place for approximately a decade, promote comprehensive reporting. TIDieR provides a 12-item checklist that ensures interventions are described in detail, including components such as who delivered the intervention, where and how it was delivered, and measures of fidelity.^13^ CERT builds upon these principles specifically for exercise-based interventions, emphasizing factors such as intensity, frequency, and individualization.^14^ Although the TIDieR and CERT guidelines are not mandatory for the publication of clinical trials, their development, along with related extensions,^15^^,^^16^ reflects a growing concern regarding the need for detailed reporting of non-pharmacological interventions in clinical trials. This concern now extends to the description of control interventions, as outlined in the *Cochrane Handbook*^17^ and has led to the creation of the Recommendations for the Development, Implementation, and Reporting of Control Interventions in Efficacy Trials of Physical, Psychological, and Self-Management Therapies (CoPPS) statement.^18^

Finally, the fourth initiative focuses on improving the fidelity of interventions, defined as the processes used to determine whether an intervention and the broader study implementation were delivered as intended by their developers. Fidelity thus captures critical information that enables researchers and even clinical practitioners to understand whether the observed effects (or lack thereof) are attributable to the intervention itself or to deviations from the intended protocol. Ensuring fidelity is essential for both internal validity (drawing accurate conclusions about effectiveness) and external validity (facilitating replication and generalizability) in clinical trials.^19^ Undoubtedly, the challenge of maintaining intervention fidelity is greater when the therapeutic approach is non-pharmacological, and even the TIDieR guideline includes two fidelity-related items under the *How well* section (items: 11.*Planed*, 12.*Actual*). However, these items alone are not sufficient, as intervention fidelity encompasses multiple domains and requires a more detailed analysis. In fact, over the past 20 years, concern has grown regarding the development of proposals for evaluating intervention fidelity,^20^ emphasizing the need to apply and refine existing frameworks and models, and to improve how fidelity is addressed.^21^ Despite these efforts, existing documents offering recommendations on intervention fidelity reflect the evolving nature of the field,^22^ and no universally accepted standard exists for reporting intervention fidelity.^23^ Moreover, the application of these proposals is not currently part of the criteria for determining whether a clinical trial is eligible for inclusion in secondary documents, such as systematic reviews (SRs) and clinical practice guidelines (CPGs).

## The lack of a more careful approach to interventions is more than a problem

It has been reported that outcomes observed in clinical trials are not consistently reproduced with the same effectiveness in real-world clinical practice,^24^ including trials of non-pharmacological therapies.^25^ In pharmacological interventions, the *what, why, how, when, where*, and *who* are typically standardized and detailed in the product leaflet. However, non-pharmacological interventions involve greater variability and require a comprehensive description to ensure reproducibility and effectiveness, which remains limited.^26^^,^^27^ Delving into the reasons for the lack of traceability between research and clinical practice often reveals a mismatch between the profiles of clinical trial participants and those of real-world patients.^28^^,^^29^ Moreover, other contributing factors are also plausible, including poor leadership engagement, lack of relative priority,^30^ insufficient infrastructure, resistance to change, and contextual differences between research and practice settings.^3^^,^^31^ Notably, one of the less frequently addressed causes of this discrepancy in the health domain is the inadequate description and fidelity of interventions, which have been previously highlighted in other fields, such as behavioral sciences^32^ and the creative arts.^19^ Thus, a lack of sufficient information about the intervention could compromise not only the replicability of studies but their translation into practice,^33^ as noted in trials involving non-pharmacological interventions.^34^, ^35^, ^36^

Specifically, Ribeiro et al^34^ emphasize that physiotherapy interventions often suffer from poor reporting of critical components, such as dosage and progression. When the intervention involves exercise, it has been noted that improving the design of randomized clinical trials to yield strong and reproducible findings regarding the health benefits of exercise training is determinant for strengthening the scientific foundation underlying the implementation and dissemination of evidence-based exercise programs.^37^ Likewise, recent studies have highlighted substantial shortcomings in the fidelity of therapeutic interventions within clinical trials of non-pharmacological interventions.^38^ These findings underscore the need for improved strategies to assess, monitor, and enhance treatment fidelity in clinical trials.^39^ Given these challenges, a profound methodological, institutional, and even cultural shift is required.

## Advancing clinical practice through improved intervention reporting and fidelity

Effective implementation strategies for translating scientific knowledge into routine clinical practice depend on multiple interrelated factors, including committed individuals who collaborate to engage relevant stakeholders, analyze workflow dynamics, and overcome barriers to delivering evidence-based care.^40^ Moreover, when research focuses on non-pharmacological intervention approaches, funding agencies should actively promote and support the integration of process evaluation studies alongside clinical trials, irrespective of the trial phase.^41^ Consequently, a deliberate and systematic approach to evaluating and refining everyday research practices, particularly in the context of non-pharmacological interventions, is necessary to ensure that scientific findings are translated into actionable knowledge and routine clinical care.

## The clinical registry as the cornerstone of a clinical trial

Discrepancies between clinical trial registration and publication have been documented in several methodological aspects, such as protocol deviations, missing data, primary outcome analyses, adjusted analyses,^42^ and blinding procedures.^43^ However, there is a lack of specific information regarding the description and fidelity of interventions.^44^ To improve this aspect within each clinical trial, the process should begin with and revolve around the clinical trial registry, as it is the first publicly available document associated with the clinical trial. If the registry includes all essential information from the initial version and is updated to reflect any modifications or clarifications made during the development and publication of related documents, including grant applications, protocols, abstracts, and preprints, much of the groundwork is already in place. In this regard, the recently updated Standard Protocol Items: Recommendations for Interventional Trials (SPIRIT) statement includes items 15a-d, which pertain to the description of the *Topic Intervention and Comparator* and their variations,^45^ offering an initial framework for the comprehensive reporting of interventions in clinical trials. Nevertheless, when reporting non-pharmacological interventions, a higher level of detail is required. Therefore, the application of reporting guidelines such as TIDieR and CERT, available through the Enhancing the QUAlity and Transparency Of health Research (EQUATOR) Network,^46^ is a necessary starting point. Moreover, when more specific guidelines exist, such as the TIDieR extension for population health and policy interventions^47^ or CERT adaptations for pelvic floor muscle training,^48^ they should be applied.

Afterward, a proper evaluation of the fidelity of the interventions, which should be reflected in all publications related to the clinical trial, can be conducted. In response to this need, several general-scope frameworks have been proposed to improve fidelity assessment, based on elements such as adherence, moderators, and the identification of core components of the intervention.^49^ In the field of rehabilitation, a specific model has been developed to ensure traceability of fidelity through a structured, linear framework,^50^ grounded in the fidelity components of the U.S. National Institutes of Health model.^20^^,^^51^ This begins with the *Design* phase, which involves defining the intervention and designing the study to evaluate it; continues with *Resourcing*, which entails preparing the necessary resources, including personnel and equipment; and culminates in *Implementation*, where the intervention is evaluated in terms of practitioner and client behaviors and interactions.^50^ Further refinements have been proposed for trials targeting specific conditions, such as multiple sclerosis, encompassing five key domains: (a) study fidelity protocol, (b) fidelity of study design, (c) fidelity of provider training (i.e., ensuring that trainers/coaches are consistent in training and experience), (d) fidelity of treatment delivery (i.e., ensuring that trainers/coaches are consistent in delivering the intervention), and (e) fidelity of treatment receipt (i.e., assessing whether participants understand and engage with the intervention as intended).^52^

As can be observed, despite sharing common goals, there is considerable heterogeneity in the nature and content of existing proposals for evaluating intervention fidelity. This underscores the need for clearer, more systematic, and globally applicable consensus on core fidelity reporting standards.^23^ In response to this gap, a new guideline, called Reporting Fidelity of Non-Drug Interventions (REFIND), is currently under development.^53^ The REFIND guideline is anticipated to serve as a standalone reporting framework for intervention fidelity.^54^

## Enhancing systematic reviews of non-pharmacological interventions is a systematic process

In 1972, Archie Cochrane published *Effectiveness and efficiency: random reflections on health services*,^55^ in which he criticized the widespread use of medical practices lacking rigorous scientific evidence. This work inspired the creation of the Cochrane Collaboration in 1993. Today, Cochrane Reviews are widely regarded as the international gold standard for high-quality, accessible, and up-to-date SRs in health research, including non-pharmacological interventions.^56^

However, Cochrane Reviews often show a lack of strong evidence supporting the efficacy of specific treatments or strategies. This was stated in 2019, when only 5.7% of Cochrane Reviews in physical therapy were deemed conclusive, a proportion lower than that observed in other medical fields. As of 2025, the proportion remains approximately 3%, while the absolute number of conclusive reviews has increased because the total number of reviews has grown.^57^ These low rates warrant further research before a final conclusion can be drawn. One potential reason for this need is the low methodological quality of many studies.^58^ In fact, addressing the risk of bias in clinical trials within physical therapy and in other non-pharmacological interventions can be particularly challenging. These include difficulties in blinding participants and providers,^59^ the development and application of sham therapies, and even the widespread use of complex, individualized therapeutic approaches.^60^ These aspects are closely linked to the adequacy of intervention descriptions and fidelity assessments, which limit researchers' and clinicians' ability to rely on Cochrane Reviews for definitive answers to specific clinical questions.^61^^,^^62^ To address this issue, it is recommended that higher-quality primary studies be developed through active collaboration and consultation with international experts and professional societies, thereby improving the reliability and applicability of evidence in non-pharmacological interventions.^63^

As described, traditional SRs often prioritize the methodological quality of clinical trials, focusing on elements such as randomization and blinding. While these factors are essential for ensuring internal validity, they do not address external validity, which depends on the replicability and clinical applicability of the interventions described. Therefore, the quality of intervention descriptions and their fidelity should be assessed, not only within individual trials, but also systematically across SRs. In this context, it is highly advisable for new clinical trials to include a comprehensive description of the interventions and an evaluation of their fidelity. This would facilitate a deeper understanding of how the effectiveness, efficacy, and efficiency of interventions depend on the quality of their description and the consistency with which they are implemented.

Since protocols that adhere to TIDieR and CERT guidelines ensure that planned interventions are well-documented, thereby facilitating subsequent fidelity assessments, SRs should include a protocolized evaluation of the alignment between registered protocols and published findings. Accordingly, it is recommended to establish a minimum threshold for such alignment as an inclusion or exclusion criterion for clinical trials included in SRs. As is routinely done with other methodological quality indicators,^64^^,^^65^ this approach could enhance the robustness of SRs and improve the clinical applicability of their findings. This proposal remains relevant even for reviews that integrate primary trial data with other sources, such as patient perspectives and expert opinion,^66^ as it contributes to a more comprehensive and reliable synthesis of evidence.

Although it exceeds the primary objective of this article, it is hypothesized that incorporating TIDieR and CERT guidelines, along with fidelity assessment, into SRs could:

− Ensure transparency by evaluating the quality of intervention reporting, enabling reviewers to identify gaps that may affect the interpretation of results and, consequently, enhance clinical applicability.
− Detect discrepancies by identifying omissions or unjustified deviations from registered protocols, thereby revealing potential gaps between internal and external validity in clinical trials.
− Guide future research by highlighting deficiencies in interventions reporting, which can inform the design of future studies, promote adherence to established reporting standards, and ultimately improve the clinical applicability of the research findings.^67^^,^^68^

## Enhancing clinical practice guidelines includes the clinical practice context

Although earlier proposals existed, CPGs are most widely recognized to have originated approximately 35 years ago.^69^ Conceptually, CPGs are statements that include recommendations intended to optimize patient care, informed by SRs of the evidence and an assessment of the benefits and harms of alternative care options.^70^ In recent years, the relevance of non-pharmacological interventions within CPGs has grown substantially.^71^ Nevertheless, as with SRs, CPGs are susceptible to becoming outdated as new evidence emerges. It has been described that 1 out of 5 recommendations may be outdated within 3 years, suggesting that waiting longer than this period to reassess a guideline may compromise its validity and clinical relevance.^72^

The overall quality of published CPGs for musculoskeletal conditions has been questioned, particularly regarding their limited clinical applicability^73^ and insufficient consideration of knowledge users’ values.^74^ While multiple factors may contribute to this lack of applicability, this section will focus on the insufficient integration of contextual factors related to the interventions recommended in CPGs, which often results in a *one-size-fits-all* approach that may limit the relevance and effectiveness of guideline recommendations in diverse clinical settings.^74^ This is particularly relevant in non-pharmacological interventions, where context plays an even more pivotal role than in pharmacological interventions.^75^

As a primary contextual factor, it is important to recognize that most CPGs have been developed within the healthcare systems of high-income countries. However, differences in resource availability, healthcare infrastructure, and cultural norms may significantly influence clinical practices in other settings.^76^ Expanding the geographical scope of guideline development to include countries with varying levels of development and resource availability could enhance the generalizability of CPG recommendations.^74^ Moreover, recommendations for non-pharmacological interventions in CPGs frequently overlook the influence of comorbidities, such as insomnia, obesity, diabetes, or anxiety, and patient-specific factors, including health literacy, social (e.g., family or peer support), financial (e.g., cost of therapies), and cultural barriers, in shaping treatment preferences and responses.^77^^,^^78^ Finally, in clinical practice, it is important to recognize the patient as an individual embedded in a unique, multifaceted context, as the biopsychosocial model posits.^79^ However, most CPGs demonstrate limited integration of users' values and preferences into the recommendation development process.^80^ This is concerning, as incorporating these variables affects the implementation of recommendations in clinical settings.^81^^,^^82^ By doing so, CPGs can contribute to the delivery of more effective, equitable, and patient-centered care.^74^

## Implications for physical therapy and other non-pharmacological disciplines

Physical therapy, as one of the most widely used non-pharmacological therapies, exemplifies the complexities associated with intervention description and fidelity. Unlike pharmacological treatments, physical therapy interventions are shaped by a multitude of factors, including professional training, cultural context, patient expectations, healthcare system variability, and the necessity to tailor treatments to the physical and psychological characteristics of individual patients,^14^ which underscore the importance of comprehensive reporting and rigorous fidelity assessment.

**Fig. 2:**
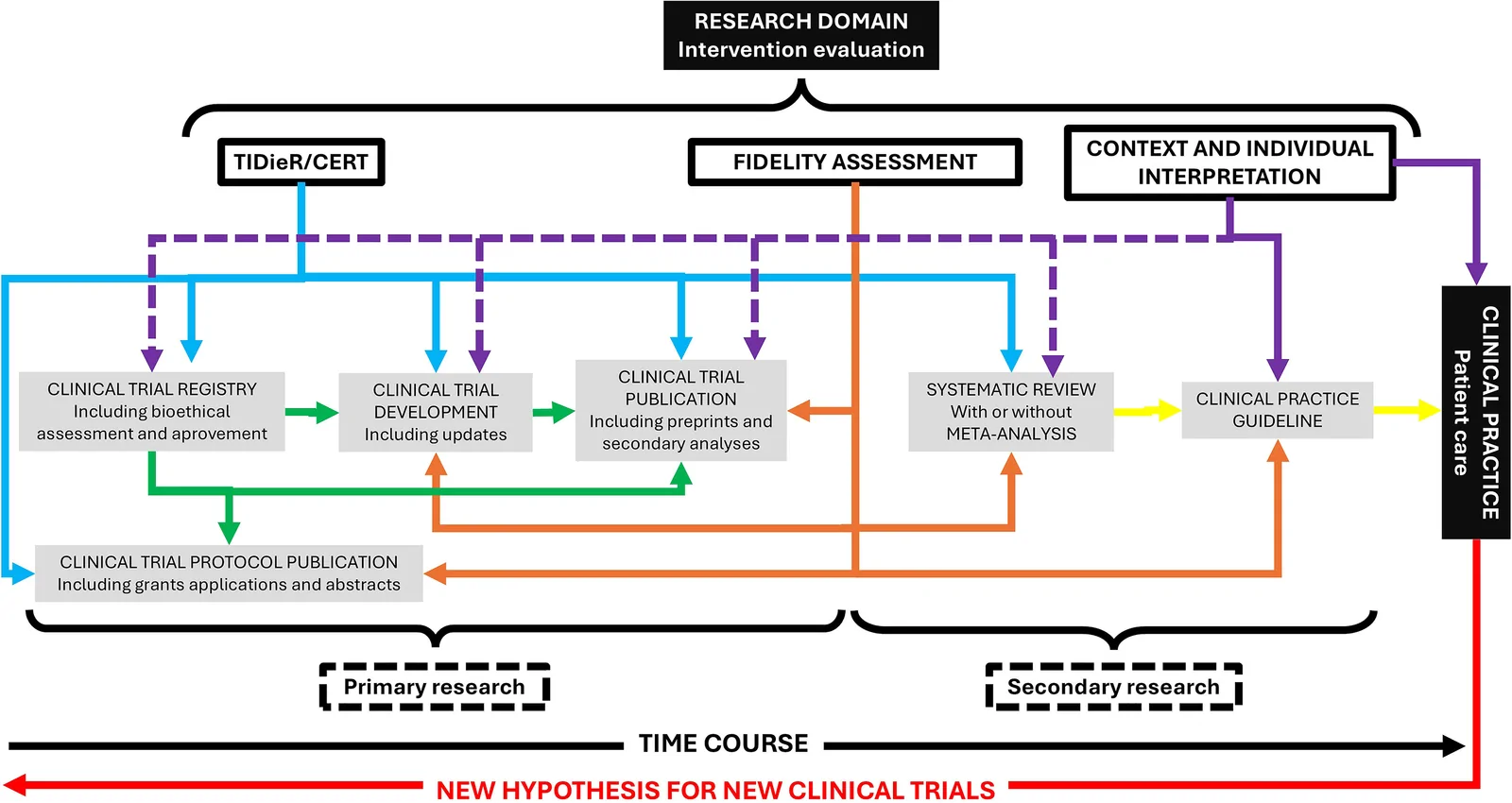
Evaluation framework for non-pharmacological interventions. Green lines indicate the influence of clinical trial registries; Blue lines indicate the influence of the TIDieR and CERT guidelines; Orange lines indicate the influence of fidelity assessment; Purple lines indicate the influence of context and individual interpretation (dashed lines represent the influence of context, specifically); Yellow lines represent the pathway from secondary research studies to clinical practice; Grey squares represent the types of documents associated with clinical trials.

As proposed in Fig. 2, the initial step to strengthen the relationship between intervention reporting and clinical practice is to improve the quality and completeness of prospective clinical trials registries. The TIDieR and CERT guidelines, specifically developed to support the reporting of non-pharmacological interventions, including control conditions, alongside CONSORT and SPIRIT guidelines, provide a structured framework to ensure sufficient detail in intervention descriptions.

Secondly, the systematic assessment of fidelity across all scientific outputs related to each clinical trial, including protocols, trial reports, secondary analyses, and SRs with or without meta-analyses, will enable a precise understanding of whether interventions were delivered as originally intended. Until a universally accepted gold standard for fidelity assessment is established, it is recommended to apply expert-consensus instruments that have demonstrated adequate psychometric properties. To summarize the essential elements of these processes, Box 1 presents a minimum reporting standard for non‑pharmacological interventions that aligns with the requirements of the main guidelines and statements.

Thirdly, CPGs should incorporate the contextual factors surrounding recommendations derived from clinical trials. These include social, organizational, and professional aspects, as well as the availability of material resources and patient-specific characteristics.

Fourthly, scientific associations, professional colleges, and funding agencies should promote improvement processes related to non-pharmacological interventions. Such initiatives would enable better oversight and quality control, from the initial conceptualization of an intervention to its eventual recommendation in clinical practice.

To operationalize these recommendations, Table 1 outlines an implementation pathway, including the minimum intervention and fidelity items and their locations by document type. It should be acknowledged that putting these proposals into practice may temporarily slow the rate of publications and, consequently, reduce the short‑term availability of high‑quality evidence. However, this trade-off is justified by the need to ensure that interventions applied in clinical settings accurately reflect those originally designed and evaluated in clinical trials. Such alignment increases the likelihood of achieving comparable outcomes in clinical practice. In other words, prioritizing quality over quantity in healthcare research may lead to more meaningful and impactful advancements. Doing less, but doing better, can ultimately result in doing more.

**Table 1:** Implementation pathway: minimum intervention and fidelity items, and where to find them.

Document type	Minimum intervention items	Minimum fidelity items	Where readers should find them
**Clinical trial registry**	Synopsis of intervention and comparator; key TIDieR fields; planned modifications/versioning.	Planned fidelity indicators; whether provider training and adherence monitoring are envisaged.	Trial registry (ICTRP/primary registry); linked protocol.
**Protocol publication (SPIRIT)**	Crosswalk to TIDieR/CERT; intervention manuals; training curricula; comparator details; detailed materials/procedures.	Domains (design, training, delivery, receipt, enactment); fidelity checklists; monitoring forms; analysis plan for adherence/fidelity; inter-rater reliability for fidelity ratings.	Protocol document.
**Clinical trial publication (CONSORT + TIDieR/CERT)**	Actual vs planned delivery; deviations and reasons; provider and site/context characteristics; intervention manuals*; training curricula*; materials*.	Observed fidelity results (provider adherence, participant adherence/receipt/enactment); strategies used to enhance fidelity; fidelity checklists*; monitoring forms*; inter-rater reliability for fidelity ratings*.	Main article; methods/results.
**Systematic review**	Protocolized appraisal of intervention reporting; threshold for inclusion/exclusion based on completeness.	Protocol-publication alignment assessment; sensitivity analyses by fidelity quality.	Systematic review methods section; protocol publication (PROSPERO, OSF…); supplementary appraisal tables.
**Clinical practice guideline**	Adaptation guidance for context (resources, culture); patient values/preferences.	Feasibility and implementability considerations; strength/consistency of fidelity evidence.	Guideline methods/appendices; evidence-to-decision tables.

*Depending on the publication requirements, these items may be included as supplementary materials.

Abbreviations: CERT: Consensus on Exercise Reporting Template; CONSORT: Consolidated Standards of Reporting Trials; ICTRP: International Clinical Trials Registry Platform; OSF: Open Science Framework; PROSPERO: International Prospective Register of Systematic Reviews; SPIRIT: Standard Protocol Items: Recommendations for Interventional Trials; TIDieR: Template for Intervention Description and Replication.

**Box 1:** Minimum reporting standard for non-pharmacological interventions.

Intervention description	TIDieR items 1–12 (who, what, why, where, when, how; materials; procedures; tailoring; modifications; How well planned/actual).
**Exercise-specific augmentation**	CERT core elements (type, dose, intensity, frequency, progression, supervision, adherence/monitoring).
**Control/comparator description**	CoPPS guidance (materials, procedures, rationale, dose, credibility).
**Fidelity**	Pre-specify provider training/competence, delivery, receipt, and enactment; report planned vs actual and strategies to enhance fidelity.

## Funding

None.

## Declaration of competing interest

The author(s) declared no potential conflicts of interest.
